# Attitudes among dermatologists regarding non-melanoma skin cancer treatment options

**DOI:** 10.1007/s12672-021-00421-w

**Published:** 2021-09-07

**Authors:** Luca Fania, Tonia Samela, Gaia Moretta, Francesco Ricci, Elena Dellambra, Mara Mancini, Francesca Sampogna, Annarita Panebianco, Damiano Abeni

**Affiliations:** IDI-IRCCS, Dermatological Research Hospital, Via dei Monti di Creta 104, 00167 Rome, Italy

**Keywords:** Skin cancer, Non-melanoma, Basal cell carcinoma, Squamous cell carcinoma, Therapy

## Abstract

Non-melanoma skin cancers include basal and squamous cell carcinoma. These tumors have become an important health issue for their high incidence and for the morbidity, especially if untreated for a long period. Over the last 20 years, therapeutic approaches for these tumours have been improved and tailored. In this survey we provided data from one hundred and ten Italian dermatologists regarding knowledge and attitude towards different therapeutic approaches on non-melanoma skin cancers. In our study, we observed that surgery and imiquimod 5% cream were the most used treatment by dermatologists for basal cell carcinoma, while, surgery was the most common treatment for cutaneous squamous cell carcinoma. Furthermore, we observed some differences regarding the prescribed therapies in the different Italian geographical areas (i.e., Mohs’ surgery and electrochemotherapy were more frequently used in Northern compared to Central and Southern Italy whereas immunotherapy was more used in Southern compared to Northern and Central Italy) and even considering the year of specialization of the dermatologists (i.e., immunotherapy with cemiplimab was prescribed mainly by dermatologists with 10–19 years of specialization). However, for locally advanced and metastatic forms of basal and squamous cell carcinoma, Hedgehog Pathway Inhibitors and anti- Programmed cell death protein antibody treatment, respectively, were used in line with the newest evolution of therapies regarding this topic. Considering the importance of skin cancers and its progressive increase in incidence, it is crucial to improve the knowledge of different therapeutic approaches among dermatologists.

## Introduction

Non-melanoma skin cancers (NMSCs) include basal cell carcinoma (BCC) and cutaneous squamous cell carcinoma (cSCC). These skin cancers have become an important health issue worldwide with an incidence that is estimated to be 18–20 times higher than that of melanoma [1]. BCC is the most frequent human cancer and accounts for 50% of all cancers in the United States [2, 3]. The reported incidence rate in Europe is 129.3 in men, and 90.8 in women per 1,00,000 person-years [4]. The reported incidence of BCC in Italy is about 100 cases per 1,00,000 person-years and accounts for 15% of all diagnosed cancers [5]. Even though the risk of death as a consequence of this cancer is not remarkable, BCC can be associated with significant morbidity, especially if the malignancy is untreated for a long period, or in case of continuous recurrences [6]. In a study on related quality of life in patients with several skin conditions, NMSC was one of the less impairing conditions from a psychosocial point of view, but, patients with NMSC reported a greater impact of symptoms than other conditions [7]. BCCs is characterized by several clinical features and different histopathological morphologies so that the clinical differential diagnosis should take into consideration a variety of diseases [5]. In fact, the diagnosis, generally supported by dermoscopy and/or confocal microcopy, should take into account advanced age, Fitzpatrick skin phototype (I–II), previous diagnosis of NMSCs, excessive sun exposure, and immunosuppression, in addition to an accurate physical examination [8]. A correct classification of BCC is of paramount importance in choosing the most appropriate treatment [9, 10]. In literature several clinical subtypes of BCC have been described but the main clinical subtypes are nodular, superficial, and morpheaform BCC [11]. Nodular BCC is characterized by a papule or a nodule with a characteristic pearly, shiny edge, and small arborizing telangiectasias. The lesion may ulcerate, but a sharp border usually is maintained [2]. Superficial BCC is characterized by a sharply circumscribed, scaly, pinkish macule, papule, or thin plaque. When a spontaneous regression occurs, it typically leaves atrophic and hypopigmented areas [2]. Morpheaform BCC consists in an indurated plaque of localized scleroderma. It is usually characterized by ivory/white, shiny, smooth, indurated plaques or depressions with poorly defined edges. This type of lesion is usually more aggressive than other forms of BCC leading to extensive local destruction [2].Nevertheless, in case of diagnostic doubt it is essential to perform a skin biopsy to conduct the histological examination [8]. A recent classification has considered BCCs as either ‘easy-to-treat’, or ‘difficult-to-treat’ [12]. The former are the most common BCCs since they account for more than 95% of these cancers whereas the latter include all locally advanced (la)BCCs, and common BCCs which, for any given reason, may pose specific management problems. Several therapeutic approaches are available to treat BCC. The correct treatment is chosen considering BCC’s features (i.e., clinical form, localization, and recurrence rates) and patient’s characteristics (i.e., comorbidity, cosmetic outcome, immunosuppression, and personal preference). The first-line treatment for ‘easy-to-treat’ BCC is surgery. Other treatment options available for BCC are cryosurgery, curettage, electrodessication, photodynamic therapy (PDT), topical therapy with imiquimod 5% cream, intralesional therapy, lasertherapy, electrochemotherapy (ECT) or radiotherapy (RT). In case of (la)BCC, which could not be treated with surgery nor radiotherapy, the recently approved targeted therapy with Hedgehog Pathway Inhibitors (HPI) (i.e., vismodegib and sonidegib) is indicated.

Traditional surgery is the most common treatment for localized BCC while Mhos surgery is a surgical procedure in which the complete excision of the tumor is examined by microscopic margin control [2]. Cryosurgery, curettage, and electrodessication are fast and cost-effective techniques utilized for the treatment of superficial BCC while PDT consists in the application of a photosensitizing agent followed by irradiation with a light source [2]. Imiquimod 5% cream is an immunotherapy approved for the treatment of superficial BCC while 5-FU, interferons, interleukin-2, and bleomycin has been utilized as intralesional chemotherapies for BCC treatment. Lasertherapy is an ablative treatment used mainly for superficial BCC, otherwise, ECT consists in the intratumoural or intravenous injection of bleomycin followed by the delivery of electric pulses to the BCC area under local or general anaesthesia [13]. Two types of RT have been utilized for the treatment of BCC that are teletherapy (external beam RT) and brachytherapy and RT is indicated in patients when surgery is contraindicated or for unresectable tumors.

cSCC is the second most common NMSC after BCC, accounting for 20% of all cutaneous malignancies and about 75% of all deaths due to skin cancer, excluding melanoma [14, 15].

The clinical features of cSCC are extremely polymorphous, and they depend also on the anatomical site and subtype [16]. In situ cSCC could be an actinic keratosis (AK) or a Bowen’s disease (BD). AK could present as a red, scaling papule or plaque generally on a sun-exposed area while BD is mostly characterized by a red, sharply demarcated, scaly plaque. Invasive cSCC is mostly detected on sun-exposed skin and generally present as a persistent ulcer or a nonhealing wound. Clinical features of cSCC depend largely on the degree of differentiation of the lesion. Well differentiated cSCC manifests as scaly nodules or plaques while poorly differentiated cSCC presents mostly as soft, ulcerated, or hemorrhagic lesions [14].

Several histopathological cSCC forms have been described, with different prognostic values. The first-line treatment for invasive cSCC is surgery, while traditional techniques (i.e., curettage, electrodessication, cryosurgery, lasers, and PDT) are available for non-invasive forms. In patients who are not candidates for surgery, ECT or RT could be the primary treatment. The study of cSCC pathogenic mechanisms have recently identified potential pharmaceutical targets allowing the development of new systemic treatments, such as immunotherapy with immune checkpoint inhibitors [i.e., Cemiplimab, an anti-programmed cell death protein (PD)-1 antibody treatment] and epidermal growth factor receptor (EGFR) inhibitors. Cemiplimab has recently been indicated for metastatic and locally advanced cSCC.

The aim of this study was to evaluate the attitude of dermatologists towards the different therapeutic options available for NMSC. For this purpose, clinicians were asked to indicate which treatments they prescribe in their daily routine clinical practice.

## Materials and methods

The data presented here have been collected in a survey conducted on a sample of dermatologists recruited by other dermatologists according to the snowball sampling procedure. The study was approved by IDI-IRCCS Institutional Ethical Committee (Approval #608-1) in line with the Helsinki Declaration standards.

The study methods have been described in detail elsewhere [17]. In brief, dermatologists who agreed to be included in the study signed a written informed consent before entering the study. Data were collected from June 16th, 2020 to August 1st, 2020.

The first part of the survey collected data from participants on sex, age, number of practicing years since dermatology residency (< 10, 10–19,  ≥ 20), geographical area in which they were currently employed (Northern, Central, or Southern Italy), type of workplace (hospital, university/research hospital, local health department, private practice).

The second part consisted in nine specific questions regarding knowledge and attitude towards different therapeutic approaches on NMSC and a question about dermatologic surgery (“Do you perform dermatological surgery for skin cancer?”). The questions concerning BCC therapeutic approach where: “out of 100 patients with new BCCs, to how many of these do you prescribe one of the following treatments?” The treatment options were: surgery, Mohs surgery, cryotherapy, PDT, Imiquimod 5% cream, curettage—laser therapy—electrocoagulation, ECT, RT, HPI. Possible answers were: never, 5, 10, 25, 50, 75, 100%.

The same questions with the same possible answers were asked for cSCC, and the treatment options were: surgery, Mohs surgery, cryotherapy, PDT, Imiquimod 5% cream, curettage—laser therapy—electrocoagulation, ECT, RT, immunotherapy with anti-PD1.

The Statistical Package for the Social Sciences for Windows, Release 26.0.0.1 (IBM Corp., Armonk, NY, USA) was used to perform all analyses. Data were described as numbers, percentages, and frequency rates. Percentages were compared using chi-square test and chi-square test for trend.

Possible answers from 5 to 100% were grouped together in order to obtain a “yes/no” dichotomous variable.

## Results

The study population included one hundred and ten dermatologists. There were 57 women (51.8%) and 53 (48.2%) men. More than 30% of them had finished Dermatology training since less than 10 years, 25.5% since 10–19 years, and 43.6% since 20 years or more. More than 60% of dermatologists reported performing dermatological surgery for skin cancers. Tables 1, 2 report the description of the study population and therapeutic approaches (“yes/no” variable) according to different levels of dermatologists’ characteristics. Concerning BCC treatment (Table 1), overall dermatologists reported performing Mohs surgery in 23.6% of cases, PDT in 66.4%, ECT in 10.0%, and HPI in 30.9% of cases. PDT was used more often by male dermatologists than female. Mohs surgery and ECT were more utilized for BCC therapy in Northern compared to Central and Southern Italy, while immunotherapy was more used in Southern compared to Northern and Central Italy. For cSCC treatment (Table 2), similarly to BCC, Mohs surgery was more utilized for cSCC therapy in Northern compared to Central and Southern Italy, while immunotherapy was more used in Southern compared to Northern and Central Italy. Moreover, dermatologists trained since 10–19 years utilized more Mohs’ surgery compared to the other groups.

**Table 1:** Description of the sample and relationship between sociodemographic features and therapeutic choices for BCC (N  =  110)

	Column %			% of used treatments for BCC			
				Mohs surgery^a^	Photodynamic therapy^b^	Electrochemotherapy^c^	Immunotherapy HPI^d^
			*Overall*	*23.6*	*66.4*	*10.0*	*30.9*
Sex	Female	51.8		28.1	**57.9**	10.5	33.3
	Male	48.2		18.9	**75.5**	9.4	28.3
Years since specialization	< 10	30.9		26.5	73.5	8.8	23.5
	10–19	25.5		35.7	67.9	10.7	50.0
	20–29	10.9		25.0	75.0	/	16.7
	≥ 30	32.7		11.1	55.6	13.9	27.8
Area	Northern	22.7		**60.0**	60.0	**20.0**	**36.0**
	Central	56.4		**17.7**	67.7	**8.1**	**21.0**
	Southern	20.9		**0.0**	69.6	**4.3**	**52.2**

^a^Mohs surgery x Area: χ^2^  =  26.63; df  =  2, p  <  0.001

^b^Photodynamic therapy x sex: χ^2^  =  3.80; df  =  1, p  <  0.05

^c^Electrochemotherapy x area: χ^2^ linear trend  =  0.04; df  =  2; p  <  0.05

^d^Immunotherapy HPI x area: χ^2^  =  8.04; df  =  2, p  <  0.05

The bold font denotes statistically significant values

**Table 2:** Description of the sample and relationship between sociodemographic features and therapeutic choices for SCC (N.110)^a^

	Dermatologists			% of used treatments for SCC			
				Mohs’ surgery^b^	Photodynamic therapy	Electrochemotherapy^c^	Immunotherapy anti-PD1^d,e^
	Column %		*Overall*	*23.6*	*22.7*	*10.0*	*20.0*
Sex	Female	51.8		28.0	22.8	5.3	19.3
	Male	48.2		18.9	22.6	15.1	20.8
Years since specialization	< 10	30.9		35.3	32.4	5.9	14.7
	10–19	25.5		21.4	28.6	21.4	42.9
	20–29	10.9		16.7	0.0	0.0	16.7
	≥ 30	32.7		16.7	16.7	8.3	8.3
Area	Northern	22.7		**40.0**	32.0	**20.5**	**24.0**
	Central	56.4		**22.6**	22.6	**4.8**	**11.3**
	Southern	20.9		**8.7**	13.0	**13.0**	**39.1**

^a^Totals may vary because of missing data

^b^Mohs surgery x area: χ^2^  =  6.60; df  =  2, p  <  0.05

^c^Elettrochemotherapy x Area: χ^2^ = 5.03; df=2, p = 0.081

^d^Immunotherapy anti-PD1 x years of specialization (group “10–19” vs all the other groups): χ^2^  =  12.88; df  =  3, p  <  0.005

^e^Immunotherapy anti-PD1 x Area: χ^2^  =  8.45; df  =  2, p  <  0.01

The bold font denotes statistically significant values

The different proportions of patients treated using each approach for BCC and cSCC are reported in Table 3. Overall, 60% of dermatologists prescribed surgery for 75% of BCC cases and 8.2% of the sample always prescribed surgery in case of BCC. Regarding Mohs’ surgery, 76.4% of dermatologists never resorted to this surgical approach, while about 15% of them prescribed it in 5% of cases, and 5.5% of them prescribed Mohs’ surgery in 10% of cases. As for PDT, 33.6% of participants never prescribed PDT, while about 23.6% of them prescribed PDT in 5% of cases. As for topical therapy with imiquimod 5% cream, 14.5% of participants never prescribed it while most dermatologists used it in 5–50% of the cases. Among dermatologists who participated in the survey, 99.1% never used lasertherapy or ECT for BCC therapy. More than three-quarters of dermatologists never prescribed RT, and less than 20% of them prescribed it only in 5% of BCC cases they treated. Almost 70% of responders never prescribed HPI for laBCC, and 24.5% of them used it only in 5% of BCC cases.

**Table 3:** % of therapies for BCC according to dermatologists (N.110)

Proportion of patients receiving one of these treatments	Never	5%	10%	25%	50%	75%	100%	Missing
	BCC	BCC	BCC	BCC	BCC	BCC	BCC	BCC
Surgery	/	2.7	2.7	5.5	20.9	60	8.2	/
Mohs’ surgery	76.4	14.5	5.5	/	/	/	/	3.6
Cryotherapy	41.8	18.2	22.7	14.5	2.7	/	/	/
Photodynamic therapy	33.6	23.6	22.7	15.5	4.5	/	/	/
Imiquimod 5%	14.5	25.5	28.2	23.6	6.4	/	/	1.8
Cur/laser/electro	99.1	0.9	/	/	/	/	/	/
Electrochemotherapy	90.0	7.3	0.9	/	/	/	/	1.8
Radiotherapy	77.3	19.1	0.9	/	/	/	/	2.7
HPI/anti-PD1	69.1	24.5	0.9	/	/	/	/	5.5

Regarding cSCC, more than half of participants performed surgery in 100% of cases, while more than three quarters 142 of them never utilized other therapies such as Mohs surgery, cryotherapy, PDT, imiquimod cream, lasertherapy and ECT (Table 4). Almost 15% of them performed RT in 10% of cases, while 11.8 and 6.4% prescribed immunotherapy with anti-PD1 in 5 and 10% of cases, respectively.

## Discussion

Over the last 20 years, therapeutic approaches for NMSCs have been improved and tailored.

Martinez and Otley [18] highlighted the aims of treatment for NMSC consisting in a complete eradication of the cancer and a preservation or restoration of normal function and cosmesis. The standard treatment approaches used, primarily and only for low-risk tumors, was cryotherapy and curettage. Otherwise, for high-risk tumors, Mohs’ micrographic surgery, excisional surgery, and RT were the most used treatments. Some years later, Griffin et al. [19] have detected that surgery was the ‘gold standard’ of treatment with high success rates for BCC and cSCC even if other less invasive treatments (i.e., topically applied therapies) were available for treatment of some cases of BCC and pre-malignant lesions. Moreover, for laBCC, authors identified systemic therapies as a paradigm shift from surgical to medical management. More recently Peris et al. [12] remarked that surgery can be considered as the first-line therapy in all types of BCCs. In addition, treatment choice for “difficult-to-treat” BCC should be discussed by a multidisciplinary tumor board, and HPI could be a valid therapeutic option.

In our survey, we observed that surgery and imiquimod 5% cream were the most used treatment by dermatologists for BCC, while surgery was the most common treatment for cSCC. As known, surgery is the first line therapy for both BCC and cSCC, as recommended by many guidelines [12, 20, 21]. About 75% of dermatologists of the sample never required Mohs surgery to treat NMSC and approximately a quarter of them required Mohs surgery in only 5–25% of cases. This low percentage can be explained by the fact that few medical Institutions in Italy have all the necessary facilities to implement this specific practice. Currently, PDT is not used by dermatologists in 33.6% for BCC cases, and in 77.3% for cSCC cases. Even in this case, the reason could be that not all medical Institutions can dispense PDT or there is not enough knowledge/confidence regarding day light PDT. However, this difference between the two types of NMSC could be explained because PDT is indicated in BCC but not in invasive forms of cSCC. Topical immunotherapy with Imiquimod 5% cream was utilized by almost 80% of interviewed dermatologists in 5–50% of BCC cases. In addition, 6.4 of them used it in 50% of BCC cases. These positive results can be explained by the manageability of this therapy and because of the unwillingness of alternative methods in many medical institutions. On the other side, only 14.5% of dermatologists never used Imiquimod for BCC treatment and about 14.5% of dermatologists used it in 25% of BCC cases. These therapeutic approaches are mostly used in ‘easy-to-treat’ forms of BCC, mainly in superficial BCC. Indeed, Imiquimod 5% cream is an immune response modifier approved for the treatment of non-facial superficial BCC, applied five times weekly for six weeks. Otherwise, adverse effects of imiquimod 5% cream including local erythema and irritation of the skin and systemic effects including fatigue, fever, and exfoliative dermatitis could limit its use. 77.3% of dermatologists of the sample never prescribed RT to treat BCC. These data are understandable because not all anatomical sites are accessible by RT and not all Institutions own this technique. However, 19.1% of them treated BCC with RT only in 5% of cases, likely to treat severe cases of BCC. Only about 8% of dermatologists prescribed ECT in 5–10% of BCC cases mainly because, as for RT, this therapeutic approach is more advisable in severe BCC cases and because not many hospital institutions have the necessary equipment. It is remarkable to note that 24.5% of dermatologists of the sample prescribed therapy with HPI to treat laBCC in 5% of cases. These results seem in line with Peris et al. [12]. In fact, authors identified 95% of BCC as “easy to treat” and 5% as “difficult to treat”.

Regarding cSCC, our data confirmed that surgery is the first-line therapy for this tumor. Indeed, more than half of participants required it in 100% of cSCC cases. It is interesting to observe that 11.8 and 6.4% of participants prescribed immunotherapy with anti-PD1 in 5–10% of cSCC cases, respectively. This reflects the possibility to prescribe Cemiplimab for the treatment of unresectable locally advanced or metastatic cSCC (Table 4).

**Table 4:** % of therapies for SCC according to dermatologists (N.110)

Proportion of patients receiving one of these treatments	Never	5%	10%	25%	50%	75%	100%	Missing
	SCC	SCC	SCC	SCC	SCC	SCC	SCC	SCC
Surgery	/	/	2.7	1.8	3.6	38.2	52.7	0.9
Mohs’ surgery	76.4	10.0	5.5	2.7	/	/	/	5.5
Cryotherapy	80.0	4.5	11.8	2.7	/	/	/	0.9
Photodynamic therapy	77.3	10.9	1.8	9.1	/	/	/	0.9
Imiquimod 5%	76.4	9.1	7.3	5.5	/	/	/	1.8
Cur/laser/electro	76.4	10.9	6.4	/	/	/	/	6.4
Electrochemotherapy	90	5.5	2.7	/	/	/	/	1.8
Radiotherapy	65.5	17.3	13.6	1.8	/	/	/	1.8
HPI/anti-PD1	80.0	11.8	6.4	/	/	/	/	1.8

It is interesting to observe that Mohs surgery and ECT were more frequently utilized for BCC and cSCC therapy in Northern compared to Central and Southern Italy, probably due to the higher availability in the first area. On the other side, immunotherapy was more used in Southern compared to Northern and Central Italy probably due to the presence of more laBCC in this region located in a lower latitude area with higher incidence of UV radiation.

Furthermore, it is interesting to note that immunotherapy is specifically prescribed in case of cSCC mainly by dermatologists with 10–19 years of specialization. It is not easy to interpret such data but it could be explained because younger dermatologists may be not confident in managing this therapy while older dermatologists may be less aware of this more recent therapy.

Altogether, our data are in agreement with the newest evolution of therapies for NMSCs.

## Conclusions

We found that the most frequently used treatments for BCC are surgery and imiquimod 5% cream and that 24.5% of dermatologists prescribed HIP therapy for laBCC. Regarding cSCC, the survey showed that surgery is the first-line therapy, while 11.8% of participants prescribed immunotherapy in less than 10% of cSCC cases. Our real-life data are in line with the newest evolution of therapies regarding this topic. Considering the importance of skin cancers and the progressive increase in incidence, it is crucial to improve the knowledge of different therapeutic approaches among dermatologists.

## Abbreviations

NMSCs: Non-melanoma skin cancers
BCC: Basal cell carcinoma
cSCC: Cutaneous squamous cell carcinoma
(la)BCCs: Locally advance basal cell carcinoma
PDT: Photodynamic therapy
ECT: Electrochemotherapy
RT: Radiotherapy
HPI: Hedgehog Pathway Inhibitors
EGFR: Epidermal growth factor receptor
